# The normal modes of lattice vibrations of ice XI

**DOI:** 10.1038/srep29273

**Published:** 2016-07-04

**Authors:** Peng Zhang, Zhe Wang, Ying-Bo Lu, Zheng-Wen Ding

**Affiliations:** 1 School of Space Science and Physics, Shandong University, Weihai, 264209, China; 2 ICQD, Hefei National Laboratory for Physical Sciences at the Microscale, University of Science and Technology of China, Hefei, 230026, China; 3 College of Physical Science and Technology, Sichuan University, Chengdu, 610041, China

## Abstract

The vibrational spectrum of ice XI at thermal wavelengths using the CASTEP code, a first-principles simulation method, is investigated. A dual-track approach is constructed to verify the validity for the computational phonon spectrum: collate the simulated spectrum with inelastic neutron scattering experiments and assign the photon scattering peaks according to the calculated normal vibration frequencies. The 33 optical normal vibrations at the Brillouin center are illustrated definitely from the *ab initio* outcomes. The depolarizing field effect of the hydrogen bond vibrations at frequencies of 229 cm^−1^ and 310 cm^−1^ is found to agree well with the LST relationship. It is a convincing evidence to manifest the LO-TO splitting of hydrogen bonds in ice crystal. We attribute the two hydrogen bond peaks to the depolarization effect and apply this viewpoint to ordinary ice phase, ice Ih, which is difficult to analyse their vibration modes due to proton disorder.

Hydrogen bonds (H-bonds) are a key factor when considering the interaction of water molecules either with other water molecules or with other substances^1^,^2^,^3^. Ice crystals possess more than 13 phase states under different conditions of temperature and pressure; in all of these states, the water molecules are linked by a network of hydrogen bonds^4^. According to the principle of residual entropy described by Linus Pauling, the arrangement of disordered protons in ordinary ice under ambient pressure, ice Ih, cannot be the equilibrium structure of the ice crystal at much lower temperatures^5^. By introducing KOH-doped defects, Kawada^6^, Tajima, Matsuo and Suga^7^ found that a first-order phase transition takes place at 72K for ice Ih and subsequently proposed the name ice XI to designate this ordered phase^8^. Neutron diffraction located the protons in ice XI and identified its orthorhombic structure with space group Cmc2_1_; all the H-bonds parallel to the c-axis are oriented in the same direction, conferring ferroelectric properties^9^,^10^,^11^. Considering the lengthy process and low temperature environment of cosmic evolution, ice in interstellar space or on remote planets should form predominantly the ice XI structure. Investigation of the spectral emission of ice at thermal wavelengths is of particular relevance due to the role of these wavelengths in the remote detection of water in interstellar space. Comparison of astronomical data with laboratory spectra can provide important information on the composition and structure of ice on planetary surfaces and in atmospheres^12^,^13^,^14^.

It is always a difficult task to assign the peaks from a given vibrational spectrum, especially with regard to lattice vibration^15^,^16^,^17^,^18^,^19^,^20^. Due to proton disordered arrangement of ordinary ice, ice Ih, to analyze the normal vibration modes theoretically is unreachable. Herein, we simulate the lattice vibration of ice XI using first principle methods. Based on new developments in simulation techniques, we present the normal vibration modes separately at the Brillouin center, providing a new perspective on the controversial issues in the translational region.

## Computing methods

The primitive cell of ice XI contains four molecules with the symmetry Cmc2_1_ in space group. Using first-principles density functional theory (DFT), the code of CASTEP is chosen to perform the geometrical optimization and phonons calculation^21^. Based on our tests, the generalized gradient approximation (GGA) function of PW91 would overestimate the inter-molecular interactions. The RPBE function^22^, on the other hand, produces a slight red-shift in the H-bonds but greater accuracy in the intra-molecular vibrations; we therefore chose the RPBE function for this work. The energy convergence and SCF tolerance should take the maximum values for phonon calculation. The energy cutoff is set at 750 eV and the K-point mesh is 6 × 6 × 3 in the reduced Brillouin zone (BZ). The norm-conserving pseudopotentials are used to calculate the phonon density of states (PDOS). The relative dielectric permittivity constants in static and high frequency limit electric fields are obtained to examine the depolarization effect.

## Results and Discussion

**Figure 1:**
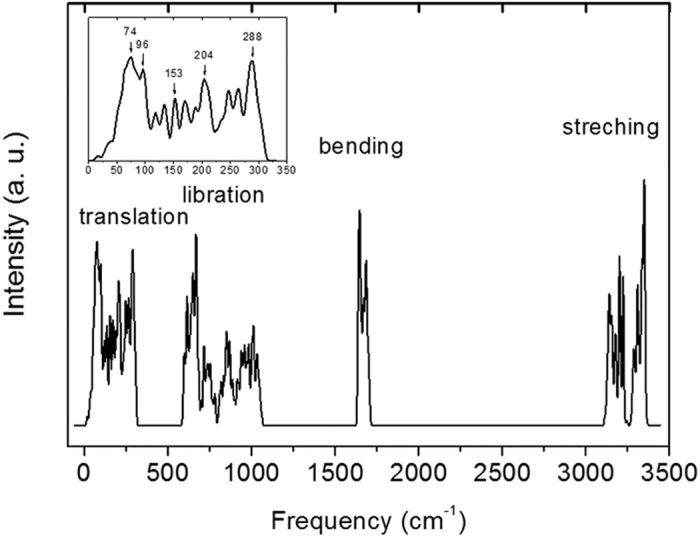
Simulation spectrum of ice XI produced with the CASTEP code. The four main vibration regions are labelled. Inset: The main peaks in the translation region.

**Figure 2:**
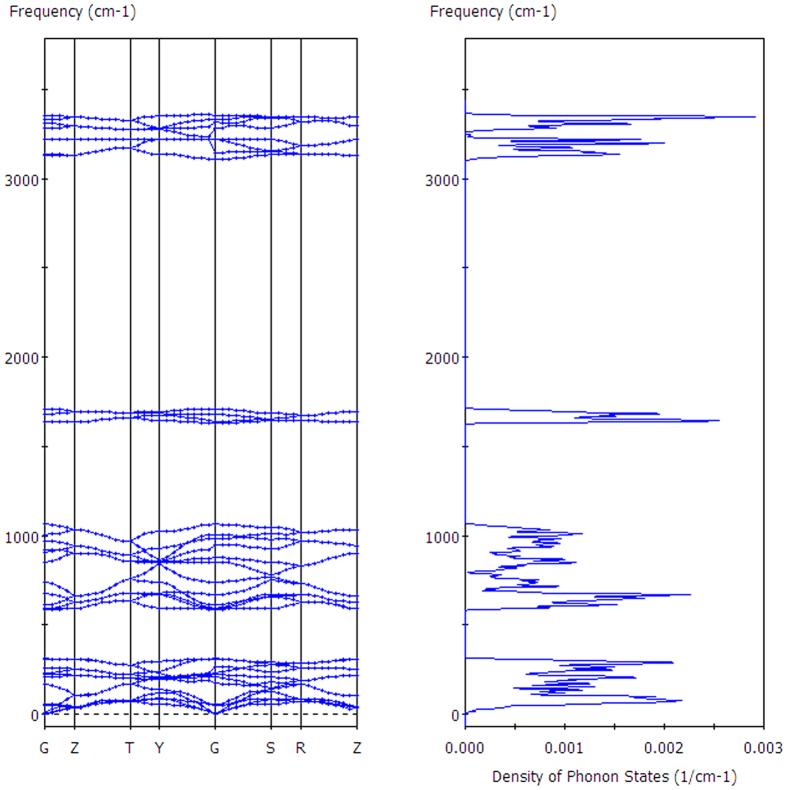
The PDOS (on the right) shows that the curve is an accumulation of the dispersion curve (on the left). Note that the vertical coordinate of the PDOS curve corresponds to the horizontal coordinate in Fig. 1 showing the vibrational frequencies.

Based on harmonic approximations, the calculated PDOS are presented in Fig. 1 with four separate main vibration regions. The ordered proton arrangements of ice XI in a 12-atom primitive cell greatly reduces the optical vibration modes, which present a more pronounced structure than the phonon spectrum of ice Ih. The results agree well with previous findings that the spectrum has the same main features as ice Ih^15^,^16^,^17^,^18^,^19^. The exchange correlation function of RPBE slightly underestimates the H-bonds, hence the spectrum peaks have a slight red-shift in the translation region and a blue-shift for the intra-molecular vibrations. We list the frequency peaks in Table 1, compared with neutron and Raman scattering data. The data from neutron scattering in the translational region are taken from ice Ih and the detailed simulation peaks are inserted in Fig. 1. Due to the structured features in the simulated spectrum, character peaks are selected with reference to experimental data and the normal modes. The normal modes at the gamma (G) point could be compared with the Raman experiments because the photon scattering may reflect the vibration signals near the Brillouin center. This table verifies the successful simulation by the first-principles DFT method. As the peaks are integrated by PDOS, it is difficult to assign one peak to a detailed vibration mode. Local vibration, such as the functional group of an organic molecule, may show a definite peak due to its flat dispersion curve. However, in the fingerprint region, lattice vibration modes accumulate into a band that it is impossible to assign a single peak to be a specific optical vibration mode. Fig. 2 shows that the integrated PDOS curve on the right is accumulated by the vibration modes, illustrated on the left as dispersion curves. We present the vibration modes in detail at the Brillouin centre, i.e., at the G point. In this case, the lattice waves have a long wavelength limit: the atoms in one primitive cell are all vibrating in relative motion while the centre of mass remains static. As the dispersion curves show, there are 3 × 12 − 3 = 33 optical branches for a 12-atom primitive cell. We therefore discuss the 33 normal modes at the G point below.

**Figure 3:**
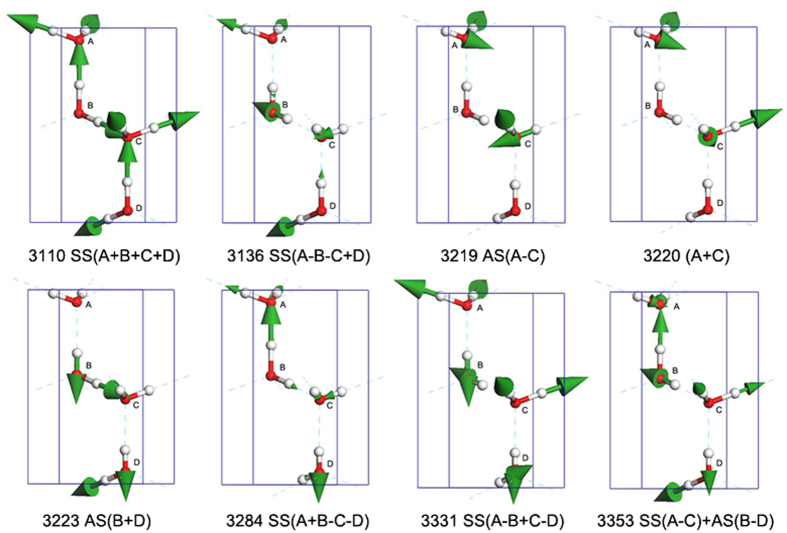
The normal vibration modes of the intra-molecular O-H stretching. The number shows the vibration frequency (cm^−1^). SS denotes symmetric stretching and AS denotes asymmetric stretching, in one H_2_O molecule. + indicates that the vibration is in phase with others of type SS or is in the same direction as others of type AS. − indicates that the vibration is out of phase with others of type SS or is in the opposite direction to others of type AS.

Figure 3 illustrates the respective vibration modes in the intra-molecular O-H stretching region. This is a side view in which the crystal axis C is along the coordinate axis Z and the normal direction bisects XY axes. The green arrows denote the simultaneous vibration direction of hydrogen atoms, in sizes proportional to the vibration amplitude. For one molecule, there are two vibration modes: symmetric stretching and asymmetric stretching. Considering the collective vibrations to be in phase or out of phase, there are 256 possible vibration mode arrangements. As the geometry is subject to the symmetry of the space group and the binding equilibrium with neighbours, there exist only 8 normal modes in the intra-molecular O-H stretching region. The notation SS represents a symmetric stretching vibration and AS represents an asymmetric stretching vibration. We also use + and − to denote whether the vibrations are in phase for SS or in the same direction for AS. It is obvious that the in phase SS for all atoms have the lowest frequencies while the AS frequencies shift higher. Shigenari *et al*. assigns the peak at 3087 cm^−1^ (Raman)/3136 cm^−1^ (IR) to SS in phase^19^. That assignment is consistent with our result of 3110 cm^−1^ (normal mode)/3139 cm^−1^ (PDOS peak). However, they also assign the Raman peaks at 3210 cm^−1^ and 3327 cm^−1^ to be in phase and out of phase SS. Moreover, their models are quite different from our analysis. There are three normal modes of AS at approximately 3220 cm^−1^. If we regard the same vibrational direction along the c-axis of molecules B and D as in phase vibration, their models seem consistent with normal modes that the mode at 3223 cm^−1^ is in phase AS while the highest vibration mode at 3353 cm^−1^ is out of phase AS, although there are different SS types between these modes. To understand the dynamic process, please find the movie of mode 3353 as Supplementary Video S1. In this movie, the crystal axes A, B and C are labelled so that we do not mark the molecules herein. It shows that molecules A and C are out of phase symmetric stretching while B and D are asymmetric stretching. Due to the inverse vibrating direction along the c-axis of B and D, we regard them as out of phase. That’s why we label this mode as SS(A-C) + AS(B-D) in Fig. 3. In the absence of neutron scattering data in this region, we take the ice Ih experiment as a reference. Li *et al*. determined two peaks at 3176 and 3312 cm^−1^ in the ice Ih spectrum to be symmetric and asymmetric stretching respectively^23^. Furic and Volovsek regarded the vibration frequencies as different sizes of cluster vibrations by fitting four peaks^17^. Overall, the vibration modes in this band are a mixture of symmetric and asymmetric stretching. We agree with the literature that the lowest peak could be attributed to the in phase SS and the peaks at higher frequencies derived from in phase SS, out phase SS, in phase AS, and out phase AS.

**Figure 4:**
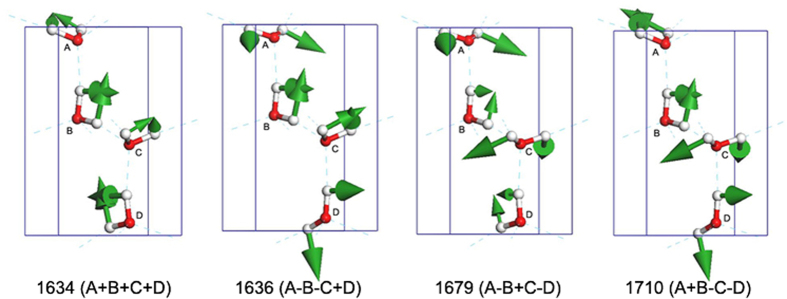
The normal modes of the intra-molecular bending vibration. The number shows the vibration frequency (cm^−1^). + and − indicate in phase or out of phase vibration with molecule A.

It is widely accepted from experimental spectra that one peak at approximately 1600 cm^−1^ is the result of the bending vibration of water. As shown in Fig. 1, the calculated spectrum shows three close peaks with a slight blue-shift at 1644 cm^−1^, 1669 cm^−1^ and 1683 cm^−1^. In fact, there are four arrangements of bending vibration mode combinations in one primitive cell, as illustrated in Fig. 4. The in phase bending for all molecules also has the lowest frequency. For example, in the third mode, in which the frequency is at 1679 cm^−1^, molecules A and C are opening while B and D are closing (see Supplementary Video S2). The neutron scattering experiment shows only one peak at 1600 cm^−1^. In the work of Shigenari *et al*., the Raman data in this region are ambiguous, resulting in an uncertain assignment. We do not discuss the broad band at approximately 2200 cm^−1^ because it is a combination so that there are not any outcome from our simulations.

**Figure 5:**
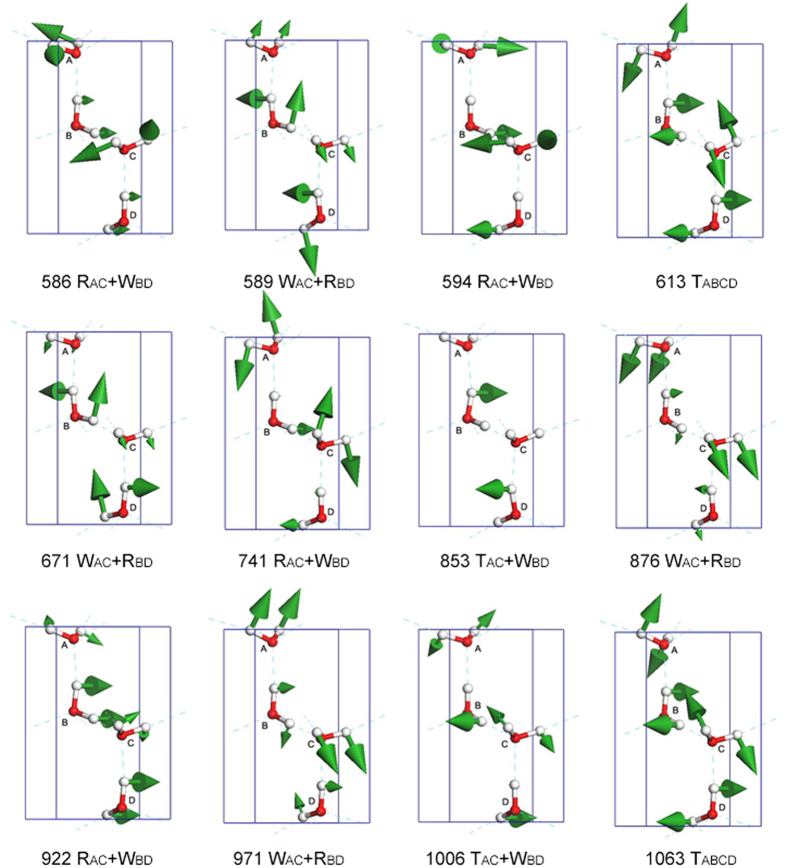
The normal modes in the libration region. The number shows the vibration frequency (cm^−1^). T, W, and R denote twisting, wagging, and rocking vibration modes. Note that the vibration frequencies at 586, 594, 741, and 922 cm^−1^ have the same symbol; 589, 671, 876, and 971 cm^−1^ are also the same. However, the vibration direction of each molecule is not the same between different normal modes.

In the libration region, the calculated frequencies of normal mode ranging from 586 cm^−1^ to 1063 cm^−1^ agree well with the neutron scattering spectrum^15^. The experimental spectrum of ice XI shows a more pronounced structure band than ice Ih in this region. Abe and co-authors presented perfect resolution of Raman scattering and clarified the 12 normal modes as different combinations of twisting, wagging and rocking vibrations relative to neighbouring molecules^16^. The arrangements of vibration modes based on our simulation are different from Abe’s work and we list each mode in detail in Fig. 5. We use the symbols T, W, R to express the twisting, wagging and rocking vibrations as described by Abe. As the four molecules have different vibrational directions, it is difficult to denote the collective vibration in phase or out of phase and thus we do not adopt the ‘−’ and ‘+’ signs in this paper. Some of the normal modes may have the same expressions: for example, the vibration frequencies at 613 cm^−1^ and 1063 cm^−1^. We use ‘T_ABCD_’ to express the twisting together of the four molecules. For the 613 cm^−1^ mode, the neighbour hydrogens keep a distance dynamically (see Supplementary Video S3). On the other hand, for the 1063 cm^−1^ mode, the hydrogen closing neighbour hydrogens alternatively (see Supplementary Video S4). Every normal mode has a unique vibration mode and the normal modes couple together throughout the whole Brillouin zone to form the libration band.

Abe *et al*. analyzed the nine vibration modes in the translation region based on group theory^18^. However, the model they used for group symmetry did not take into account of the position of the hydrogen and the vibration modes may not actually be reflective of the true situation. The peaks from experimental data that are an accumulation from PDOS, as illustrated from Fig. 2, do not necessarily point to an exclusive vibration mode. As they admitted that the data are not reproducible and some peak assignments maybe tentative, some data may be experimental phenomena. In fact, our calculated vibration frequencies are very similar to the lattice dynamical simulation by Bosi *et al*. who treated water molecules, including hydrogen atoms, explicitly^24^. This approach produces two dispersion curves at frequencies above 300 cm^−1^, instead of only one curve as derived from Abe’s model. Due to the adoption of different systems to produce the vibration modes, we do not compare the normal modes with Abe’s suggestions on an individual basis, although some maybe identical.

**Figure 6:**
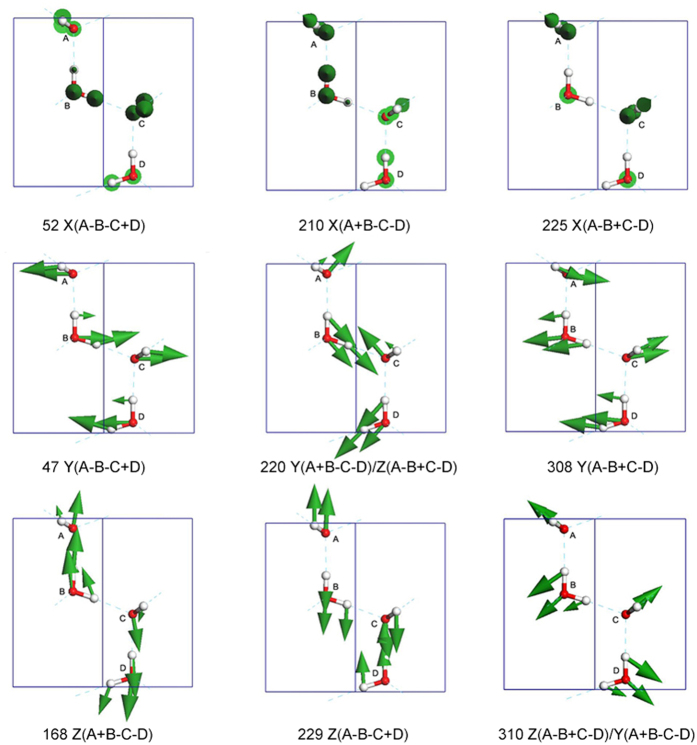
The normal modes in the translational region. The number shows the vibration frequency (cm^−1^). The side view corresponds to the YZ plane, i.e., the bc plane in the unit cell. The vibration axes are labelled X, Y, or Z. + and − indicate the directionality of movement, either the same or a different direction, relative to molecule A. Note that the vibration modes at 220 cm^−1^ and 310 cm^−1^ are in the YZ plane.

Figure 6 presents the nine normal modes in detail. The side view is in the YZ plane, i.e., the bc plane in the unit cell. In the translational region, there are only the inter-molecular vibration modes. Row 1 lists the three normal modes vibrating along the X axis. We use plus or minus signs to indicate the simultaneous vibrational directions of the four molecules. The normal mode of vibration frequency at 52 cm^−1^ labelled X (A−B−C+D) indicates that the vibration is bending along X axis, with the BC molecules at a dihedral angle. This is a skeleton deformation without H-bond stretching hence the frequency is much lower. The vibration mode at 210 cm^−1^ shows twisting along the Z axis and the vibration mode at 225 cm^−1^ denotes ABC and BCD wagging. Row 2 lists three normal modes vibrating approximately in line with the Y axis. The frequency at 47 cm^−1^ is also a skeleton deformation along the Y axis without any H-bond change, whereas the mode at 308 cm^−1^ clearly shows the H-bond vibrating along the Y axis. The normal mode at 220 cm^−1^ is an H-bond stretching in the YZ plane. The figure clearly shows that the H-bond components along Y and Z axis are vibrating out of phase. A similar case can be found at 310 cm^−1^ where the H-bond components along the Z and Y axes are vibrating in phase. The two other normal modes along the Z axis are 168 cm^−1^ and 229 cm^−1^. For the mode at 168 cm^−1^, only a rotation of the H-bond between molecules B and C is observed; this frequency is therefore the lowest. In addition, we disagree with Profeta and Scandolo that there is no peak at approximately 150 cm^−1^ for ice XI^20^.

The modes at 229 cm^−1^ (see Supplementary Video S5) and 310 cm^−1^ (see Supplementary Video S6) are of particular interest. Analysis of the vibration modes along the Z axis shows that the stretching of the H-bonds of A-B and C-D are out of phase at 229 cm^−1^ but are in phase at 310 cm^−1^. The proton ordering of ice XI results in ferroelectric properties along the Z axis. Considering the vector of polarization intensity, there is no net change for the 229 cm^−1^ vibration because the changes of the two H-bonds are cancelled out. However, for the 310 cm^−1^ vibration, the vector of polarization intensity changes with the vibration and the depolarization effect must therefore be taken into account. The depolarization field would increase the restoring force and raise its frequency. Table 2 presents the simulated dielectric permittivity constant tensors for both the static and high limit frequency electric fields. The well-known LST relationship for ionic crystals is described by the equation below^25^,


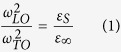


in which *ω*_*LO*_, *ω*_*TO*_ denote longitudinal waves and transverse waves, respectively, and *ε*_*s*_, *ε*_*∞*_ represent the relative static and high limit frequency dielectric constants. With regard to the ratio of two dielectric constants along Z axis, the equation becomes,


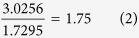


If we calculate the square of 310 over 229, the ratio is,


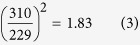


Comparing these two results above, the discrepancy is,


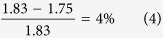


Given this result, the LST relationship seems to works in this case if we regard the vibration frequencies at 229 cm^−1^ and 310 cm^−1^ as TO and LO waves. In fact, they are actually vibrations along the c-axis with or without changes in the polarization intensity. If we take these two modes to be LO and TO splitting, our result is identical to the hypothesis by Klug and Whalley^26^ that the LO-TO splitting maybe extend to 80 cm^−1^.

When we compare the square ratio of the two H-bond peaks,


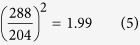


And the discrepancy is 12%. It should be noted that the spectrum of neutron scattering is a collective of PDOS throughout the whole BZ. The peaks reflect the integration of vibration modes illustrated as dispersion curves in Fig. 2. Table 1 presents the agreement of vibration peaks with neutron scattering. We do not attribute the peaks to specific vibration modes, especially in the translational region, due to the overlap of the dispersion curves. However, the Raman scattering could collect vibration signals near the Brillouin centre, which enables comparison of the normal modes with Raman experiments. In this case, it makes no sense to compare the LST relationship with the two H-bond peaks from neutron scattering, which are in fact accumulations of the vibration signals. Categorizing the vibration modes into two groups, one group with a depolarization effect while the other without, the summed H-bond vibrations should take shape of two bands. It is a controversial issue to discuss the two distinct H-bonds in the neutron scattering spectrum of ice^26^,^27^,^28^,^29^ and our previous work has refuted the strength of models incorporating two H-bonds^30^,^31^. However, the derivation of the two H-bond peaks remains uncertain and a consideration of the depolarization effect would shed light on their physical source.

## Conclusions

In summary, the PDOS of ice XI are simulated using a first-principles DFT method. Based on successful results compared with neutron and Raman scattering spectra, we analyze the specific normal modes. These 33 modes are derived directly from the *ab initio* quantum mechanical theory without the addition of any empirical model. Herein, we can see that the derivation using the symmetry of group theory has its limits and that any tentative assignments may not match the accuracy of physical data. We put forward a combined method to analyze the vibration spectrum as that: The neutron scattering spectrum could be compared with PDOS peaks, and the photon scattering spectrum could be assigned according to normal modes get from first principles simulation. This dual-track approach can verify the validity and present scientific conclusions for vibration spectrum analyzation.

The spectrum of ice XI is very similar to ordinary ice, Ih, in agreement with the literature. The ordered arrangement of the protons reduces the vibration modes, which demonstrate distinct features compared with ice Ih. The depolarization effect in the translational region for the frequencies of 229 cm^−1^ and 310 cm^−1^ is found to be in agreement with the LST relationship. The collective effects of the depolarization field in the translational region should be responsible for the two distinct H-bond peaks found in the neutron scattering spectrum. And this should be applied to other phase of ice especially for ice Ih, which is difficult to analyse their normal modes due to proton disorder.

## Additional Information

**How to cite this article**: Zhang, P. *et al*. The normal modes of lattice vibrations of ice XI. *Sci. Rep.* **6**, 29273; doi: 10.1038/srep29273 (2016).

## Supplementary Material

Supplementary Legends

Supplementary Video S1

Supplementary Video S2

Supplementary Video S3

Supplementary Video S4

Supplementary Video S5

Supplementary Video S6

## Figures and Tables

**Table 1:** Comparison of the simulated results with experimental data.

PDOS	Neutron scattering^15^	Normal modes	Raman scattering^16^,^17^,^18^,^19^
74	57	47	46
		52	50
96	106		
153	152	168	170
204	227	210	231
		220	237
		225	
		229	
288	303	308	283
		310	326
613	632	586	600
667		589	630
		594	
		613	
		671	
715	724	741	685
848	800	853	800
		876	815
957	924	922	950
1009		971	985
1032		1006	1020
		1063	1070
1644	1600	1634	1520
1669		1636	1600
1683		1679	1680
		1710	1780
3139	3176	3110	3087
3200		3136	3210
3221		3219	
		3220	
		3223	
3346	3312	3284	3327
		3331	3414
		3353	

The column ‘PDOS’ contains the characteristic vibrational peaks (cm^−1^) from the calculated vibrational spectrum. The neutron scattering peaks are listed in the second column. In the third and fourth columns, the 33 optical normal vibrational modes at G point are compared with Raman scattering data.

**Table 2:** Calculated relative static and high frequency dielectric permittivity constants of ice XI.

Optical Permittivity (f > infinity)			DC Permittivity (f = 0)		
1.7295	0	0	3.0256	0	0
0	1.7231	0	0	2.4050	0
0	0	1.7273	0	0	2.7938

The first row is the ε_∥_ along the c-axis and the next two rows are the ε_⊥_components.
