# Supplementary material for: The normal modes of lattice vibrations of ice XI
Source: Sci Rep. 2016 Jul 4;6:29273. doi: 10.1038/srep29273 (PMC4931684; doi:10.1038/srep29273)
Supplement: Supplementary Legends [file srep29273-s1.pdf]

The normal modes of lattice vibrations of ice XI

Peng Zhang[1]*, Zhe Wang[2], YingBo Lu[1], ZhengWen Ding[3]

[1]School of Space Science and Physics, Shandong University,Weihai, 264209, China

[2]ICQD, Hefei National Laboratory for Physical Sciences at the Microscale, University of Science and Technology of China, Hefei, 230026, China

[3]College of Physical Science and Technology, Sichuan University, Chengdu, 610041, China

Video Legends

S1.

The vibration mode at 3353 cm^{-1}. Molecules A and C demonstrate intra-molecular symmetry stretching in out of phase while B and D is asymmetry stretching. The stretching directions along c-axis of B and D are opposite. And we take them as out of phase. This mode is labelled as SS(A-C)+AS(B-D) in Fig. 3.

S2.

The intra-molecular bending vibration at 1679 cm^{-1}. Molecules A and C are opening while B and D are closing. We use ＋ and － to indicate in phase or out of phase vibration with molecule A. Thus it is labelled as A-B+C-D in Fig. 4.

S3.

The vibration mode at 613 cm^{-1} showing that the neighbour hydrogens always keep a distance while vibrating dynamically. The individuals vibration is twisting. This mode is named as T_{ABCD} in Fig. 5.

S4.

The dynamic vibrating at 1063 cm^{-1} is very similar to 613 cm^{-1}. The difference could be found that the neighbour hydrogens are connecting alternatively although the label are the same in Fig. 5.

S5.

The hydrogen bonding vibration at 229 cm^{-1}. It is clearly shows that while the hydrogen bond between A and B is stretching, the hydrogen bond between C and D along Z-axis is shrinking. The sum effect of dipole moments changing are cancelled out.

S6.

The high frequency vibration of hydrogen bonding at 310 cm^{-1} shows that the two hydrogen bonds along Z-axis are stretching in phase. The sum effect of dipole moments are changing dynamically. Note that the hydrogen bonds between B and C are changing too. Since the dipole moments along the Y-axis are opposite between neighbour layers. The sum effect in the Y-axis are cancelled out.
