# Supplementary figures and images for: The normal modes of lattice vibrations of ice XI
Source: Sci Rep. 2016 Jul 4;6:29273. doi: 10.1038/srep29273 (PMC4931684; doi:10.1038/srep29273)

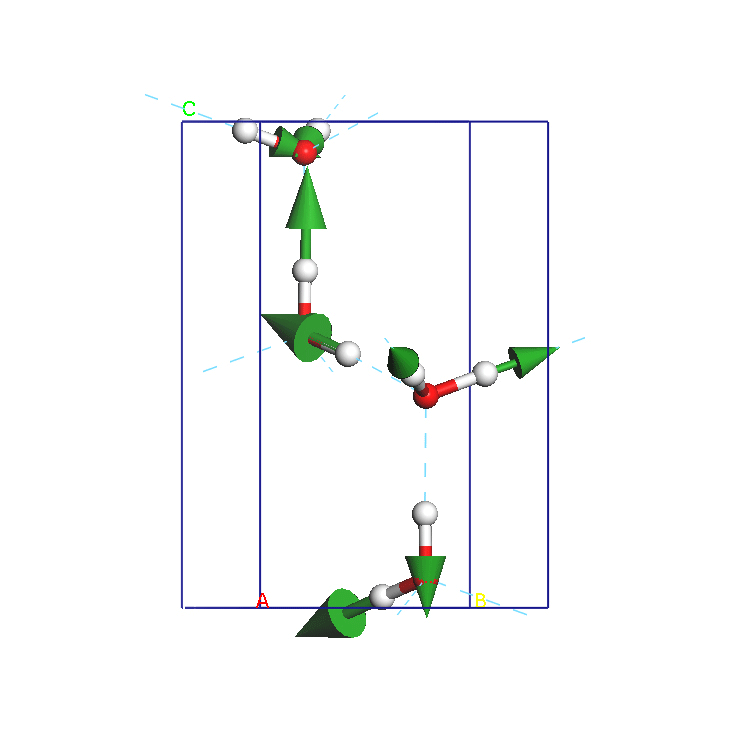

Supplement: Supplementary Video S1 [file srep29273-s2.gif]

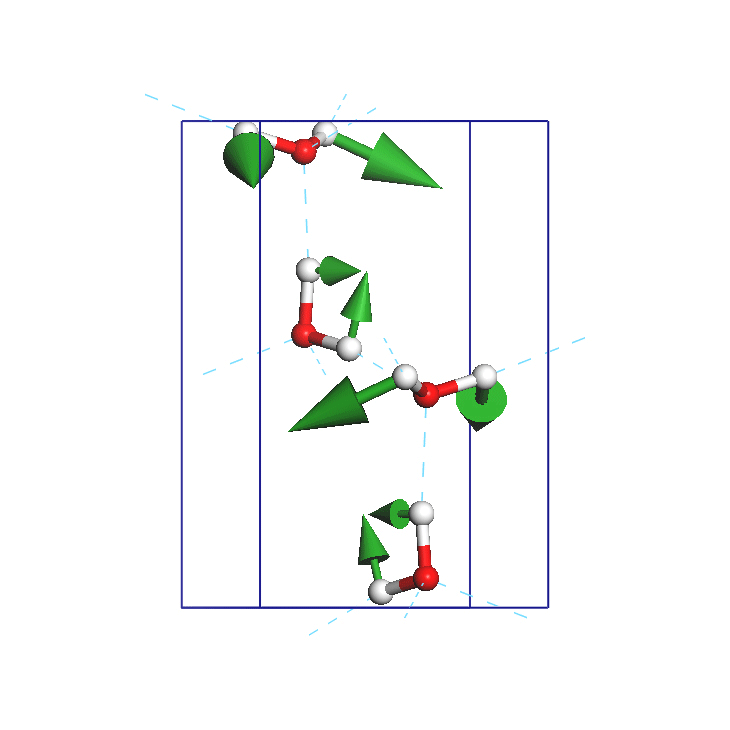

Supplement: Supplementary Video S2 [file srep29273-s3.gif]

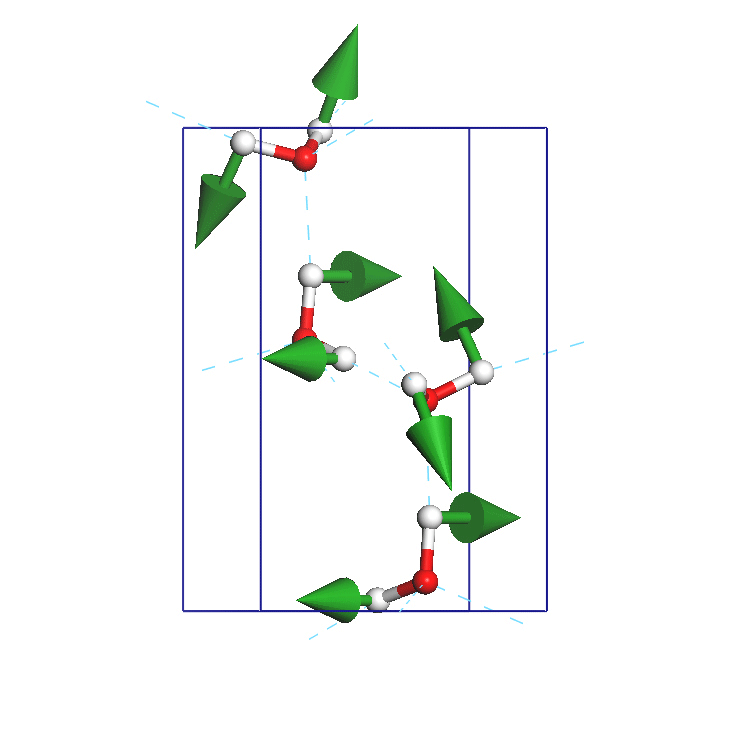

Supplement: Supplementary Video S3 [file srep29273-s4.gif]

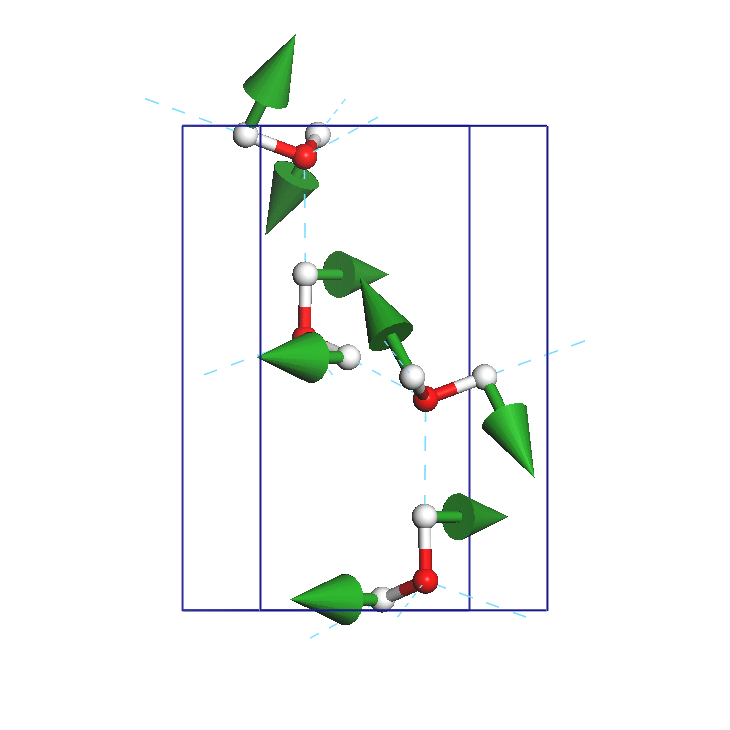

Supplement: Supplementary Video S4 [file srep29273-s5.gif]

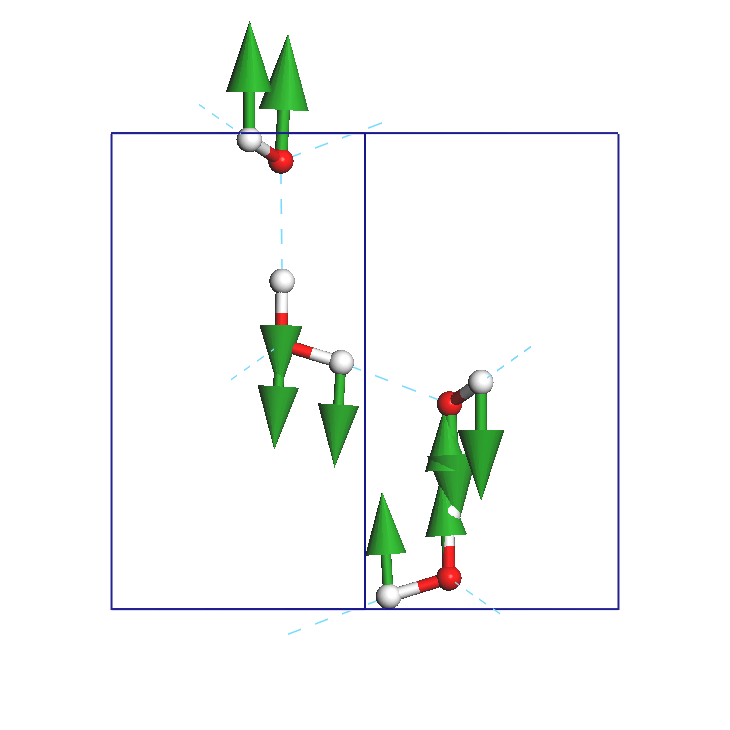

Supplement: Supplementary Video S5 [file srep29273-s6.gif]

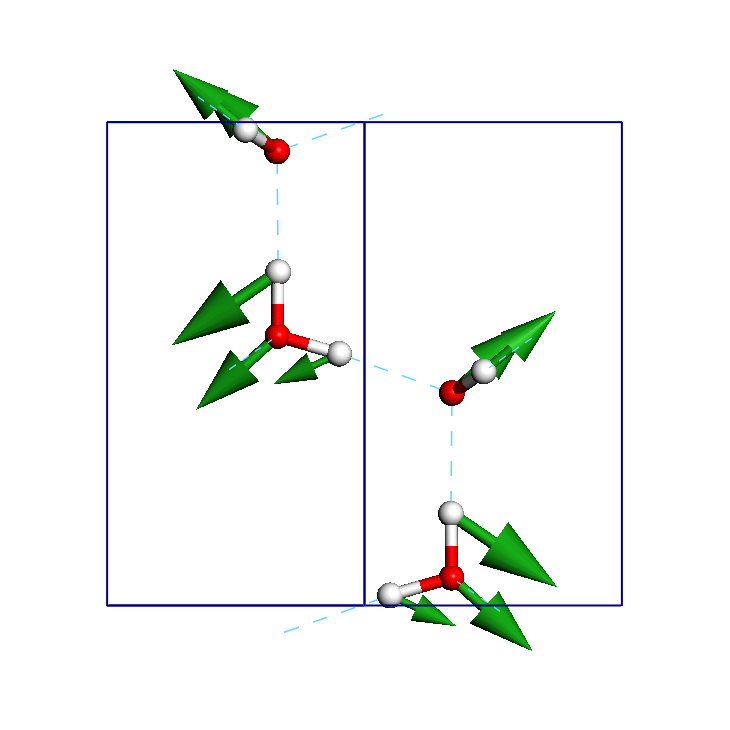

Supplement: Supplementary Video S6 [file srep29273-s7.gif]
